# Reduced lumbar myofascial mobility in chronic low back pain quantified using MR elastography-based slip interface imaging

**DOI:** 10.1186/s41747-026-00812-0

**Published:** 2026-10-01

**Authors:** Emi Hojo, Daiki Ito, Yi Sui, Xiang Shan, Phillip Rossman, Armando Manduca, Garret M. Powell, Kai-Nan An, Richard L. Ehman, Sanjeev Nanda, Brent A. Bauer, Ziying Yin

**Affiliations:** 1 Department of Radiology, Mayo Clinic, Rochester, MN USA; 2 Department of Physiology and Biomedical Engineering, Mayo Clinic College of Medicine, Rochester, MN USA; 3 Orthopedics Research, Mayo Clinic College of Medicine, Rochester, MN USA; 4 General Internal Medicine, Mayo Clinic College of Medicine, Rochester, MN USA

**Keywords:** Biomarker, Lumbar multifidus, Magnetic resonance elastography, Repeatability, Slip interface imaging

## Abstract

**Objectives:**

Restricted sliding at lumbar myofascial interfaces may contribute to chronic low back pain with myofascial pain features (CLBP-MP). We aimed to evaluate magnetic resonance elastography (MRE)-based slip interface imaging (SII) to quantify lumbar intermuscular myofascial mobility in healthy volunteers (HV), assessing repeatability, and comparing CLBP-MP patients with matched HV.

**Materials and methods:**

MRE/SII was performed in 20 CLBP-MP patients and 41 HV. SII-derived maximal normalized displacement discontinuity (Dnorm) was analyzed at the quadratus lumborum–erector spinae (QL–ES) and ES–multifidus (ES–M) interfaces at L3–L4, computing mobility indices. Repeatability was assessed *via* within-subject coefficient of variation (wCV) and Bland–Altman analysis. In HV, associations with age/sex/body mass index (BMI) were tested. Group differences were evaluated by comparing patients with matched HV and by stratifying patients by “pain, enjoyment, and general activity” (PEG) score using one-way ANOVA. Associations with PEG were evaluated using Pearson correlation.

**Results:**

Repeatability was excellent (mean wCV < 10% for within- and between-day). In HV, myofascial mobility differed by anatomic interface and vertebral level but showed no association with age, sex, or BMI. At L4 ES–M, mobility showed a significant group effect (F(2,37) = 13.23, *p* < 0.001), with lower mobility in moderate-to-severe *versus* mild pain (*q* = 0.023) and *versus* HV (*q* < 0.001), and correlated negatively with PEG (*r* = −0.51, *p* = 0.021).

**Conclusion:**

Pain-related reductions in localized lumbar intermuscular myofascial mobility in CLBP-MP were shown. MRE-based SII is a repeatable quantitative technique, supporting its potential as an early-stage biomarker for characterizing lumbar myofascial mobility and guiding multicenter studies.

**Key Points:**

- ***Question*** Lumbar myofascial interface sliding is essential for normal movement, but objective quantification of sliding dysfunction in chronic low back pain remains limited.
- ***Findings*** SII showed high repeatability and reduced L4 erector spinae-multifidus mobility associated with greater pain severity in chronic low back pain patients.
- ***Relevance statement*** MR elastography-based SII offers repeatable, noninvasive measurement of lumbar myofascial mobility and shows pain-related localized restriction in chronic low back pain with myofascial features, supporting objective characterization of inter-muscular myofascial mechanics and exploratory biomarker development.

**Graphical Abstract:**

**Figure:**
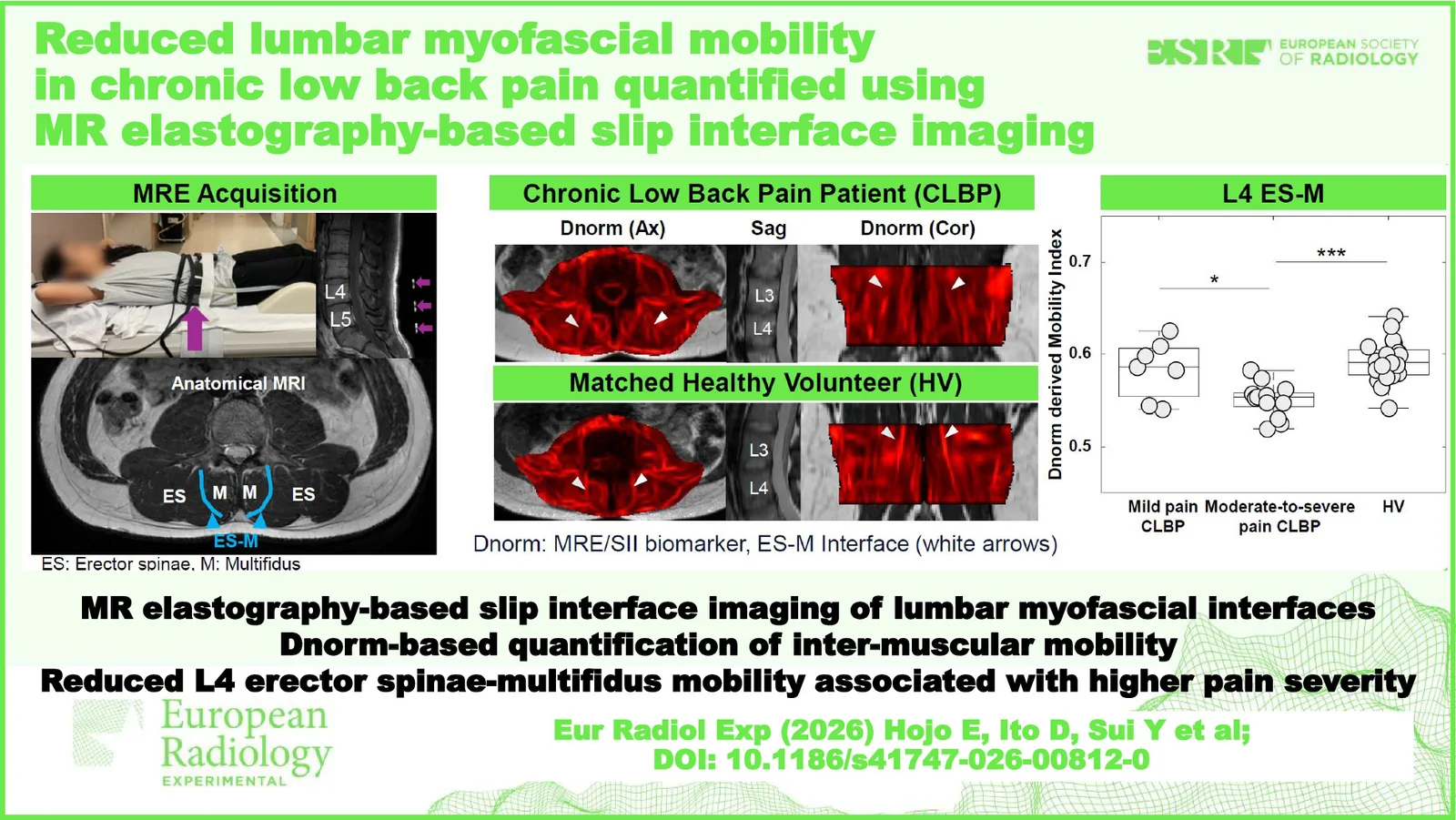

## Background

Chronic low back pain (CLBP) is a leading cause of disability worldwide [1]. Myofascial pain (MP), defined as pain from muscle-fascia complex dysfunction with myofascial tension and clinically identifiable tender points or trigger-point-like tenderness [2], affects up to 90% of individuals with CLBP [3]. Despite its high prevalence, objective and quantitative methods for assessing myofascial mechanical properties and associated abnormalities are lacking [4], and diagnosis relies on palpation and patient self-reported symptoms [5], highlighting the need for standardized assessment tools.

A mechanism underlying chronic low back pain (CLBP) with myofascial pain features (CLBP-MP) is recurrent inflammation from muscle misuse, causing myofascial adhesions that restrict inter-layer sliding, alter load distribution, and contribute to pain [6]. Ultrasound studies have demonstrated reduced thoracolumbar fascial gliding and altered shear strain during lumbar muscle activation *in vivo* [7]. Furthermore, CLBP has been associated with structural and mechanical alterations in the erector spinae (ES), multifidus (M) [8–13], and quadratus lumborum (QL) [14], which may increase mechanical coupling and limit sliding between adjacent muscle compartments. These findings motivate investigation of inter-muscular interfaces as potential sites of mechanical restriction in CLBP-MP.

To quantify myofascial interface mobility, we developed a magnetic resonance elastography (MRE)-based slip interface imaging (SII) biomarker, the maximal normalized displacement discontinuity (Dnorm) [15], which provides a noninvasive measure of relative motion across two adjacent tissue layers. SII leverages MRE-derived displacement fields to estimate the maximal discontinuity in cyclic displacement across an interface, thereby quantifying interfascial sliding. Developed to quantify brain tumor tissue adhesion [16, 17], SII has demonstrated capability for visualizing and quantifying inter-muscular interface mobility in skeletal muscles [15, 18]. Preliminary pilot data showed reduced mobility at the ES–multifidus (ES–M) interface in CLBP-MP compared with healthy volunteers (HV) [15]. This study evaluates a larger cohort and focuses on repeatability, healthy variability, and pain-related group differences.

The objectives were: (1) to establish the test–retest repeatability of Dnorm; (2) to characterize inter-muscular myofascial mobility in HV and its associations with age/sex/body mass index (BMI); (3) to compare myofascial mobility between CLBP-MP and age/sex/BMI-matched HV and to investigate correlations with self-reported measures. We hypothesized that reduced myofascial mobility in CLBP-MP and that mobility measures would correlate with clinical symptom severity. Assessments focused on the QL–ES and ES–M interfaces at L3 and L4, which are commonly affected in CLBP [6, 14]. Patients were stratified by pain severity, a clinically relevant contributor to CLBP heterogeneity.

## Materials and methods

### Study design and participants

**Fig. 1:**
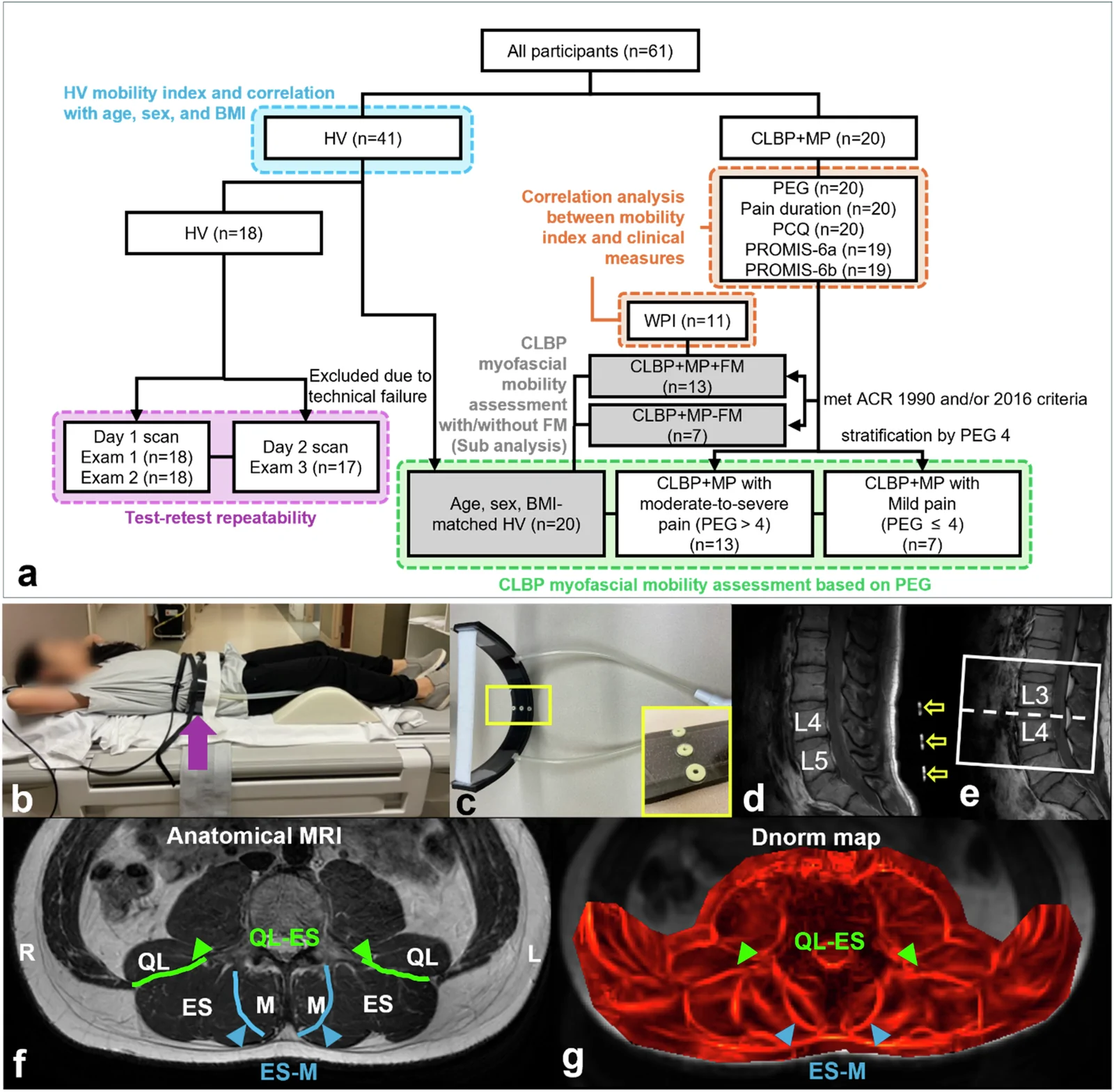
**a** Study flow diagram showing enrollment of HV and patients with chronic low back pain with myofascial pain features (CLBP-MP). Patients completed questionnaires assessing pain severity, pain duration, pain catastrophizing, sleep disturbance, and physical function, and were stratified by pain, enjoyment, and general activity (PEG) score (≤ 4 *versus* > 4). Patients meeting American College of Rheumatology criteria for fibromyalgia (FM) underwent additional assessment using the Widespread Pain Index. A subset of HV completed repeat scans for test–retest analysis, and an age-, sex-, and BMI-matched healthy volunteer subgroup was used for patient comparisons. Sub-analyses were also performed comparing patients with and without FM to HV. Numbers indicate participants included in each analysis; minor variations reflect missing questionnaire data or criteria-specific availability. **b** Supine imaging setup with the belt driver positioned over the L4–L5 region (purple arrow). **c** Customized belt driver with magnetic resonance imaging (MRI)-visible fiducial markers (yellow outline) used for anatomical localization. **d** Sagittal localizer image showing fiducial markers (yellow arrows) identifying the L4–L5 anatomical level. **e** Sagittal image indicating axial slice coverage at L3 and L4 (white rectangle); dashed line marks the corresponding axial slice level shown in **f** and **g**. **f** Axial anatomical image showing right and left QL, ES, and multifidus muscles with inter-muscular interfaces outlined. **g** Corresponding Dnorm map demonstrating high intensity contrast along the QL--ES and ES–M slip interfaces, indicating relative inter-muscular mobility. Dnorm values sampled along the segmented interfaces were averaged to compute a single mobility index for each interface region. ACR, American College of Rheumatology; BMI, Body mass index; CLBP-MP, Chronic low back pain with myofascial pain feature dnorm maximal normalized displacement discontinuity; ES, Erector spinae; FM, Fibromyalgia; HV, Healthy volunteers; L, Left; M, Multifidus; PCQ, Pain catastrophizing questionnaire; PEG, Pain enjoyment and general activity; PROMIS-6a, Patient-reported outcomes measurement information system sleep disturbance short form; PROMIS-6b, Patient-reported outcomes measurement information system physical function short form; QL, Quadratus lumborum; R, Right; SII, Slip interface imaging; WPI, Widespread pain index

This prospective study was approved by the Institutional Review Board (IRB no. 22-007647), with written informed consent obtained from all participants. Sixty-one participants (20 CLBP-MP and 41 HV) were recruited at Mayo Clinic (Rochester, MN, USA) between October 1, 2022 and August 30, 2025. Study flow and subgroup classification are shown in Fig. 1a.

Inclusion criteria for patients were: age ≥ 18 years; BMI ≤ 30 kg/m^2^; CLBP ≥ 3 months with predominant pain attributed to clinically identifiable MP; and without manual or myofascial treatment targeting the area of interest within the preceding three months. Because MP and fibromyalgia (FM) frequently coexist [18–20], patients meeting the American College of Rheumatology 1990 [21] and/or 2016 [22] criteria for FM were eligible if lumbar MP was also diagnosed as the primary source of pain, reflecting the CLBP-MP heterogeneity. Exclusion criteria included severe back injury, severe spinal or hip osteoarthritis, and skin injury or dermatologic conditions over the region-of-interest.

Inclusion criteria for HV were: age ≥ 18 years; BMI ≤ 30 kg/m^2^; and no history of low back pain or other musculoskeletal disorders. A subset of HV was matched to patients by age (± 2 years), sex, and BMI (± 2 kg/m_2_).

Immediately before magnetic resonance imaging (MRI), all patients completed questionnaires including: the pain, enjoyment, and general activity (PEG) scale, assessing pain intensity and functional interference on a 0-to-10 scale, with higher scores indicating greater severity [23]; pain duration; the pain catastrophizing questionnaire (PCQ), a thirteen item self-report instrument on a 0-to-52 scale, where higher scores indicate greater pain-related catastrophizing across the domains of rumination, magnification, and helplessness [24]. Sleep disturbance and physical function were assessed using the Patient Reported Outcomes Measurement Information System (PROMIS)-6a and PROMIS-6b questionnaires, respectively [25, 26]. Each consists of six items scored on a 1-to-5 scale with a maximum score of 30. Higher PROMIS-6a scores indicate worse sleep disturbance [25], whereas higher PROMIS-6b scores indicate better physical function [26]. For patients with comorbid FM, pain distribution was assessed using the widespread pain index (WPI), which ranges from 0 to 19, with higher scores indicating pain in a greater number of body regions [27].

Three separate investigations were conducted: (1) test–retest repeatability in a subset of 18 HV; (2) characterization of myofascial mobility and its correlations with age/sex/BMI in 41 HV; and (3) comparison of mobility indices between patients and 20 matched HV. Patients were stratified by PEG into mild (PEG ≤ 4) or moderate-to-severe pain (PEG > 4) [23, 28], with comparisons among these groups and HV. Exploratory analyses further evaluated the effects of FM comorbidity and clinical phenotype heterogeneity (*i.e*., variation in FM status and pain severity). Patients were stratified by FM, with comparisons among HV, CLBP-MP without FM, and CLBP-MP with FM. Within the patient cohort, correlations were assessed between mobility indices and the clinical assessments described above. PEG associations with mobility indices were further evaluated separately in FM and non-FM subgroups. Given limited subgroup sizes, these analyses were considered descriptive and exploratory and not intended for subgroup validation.

### MRI/MRE protocol

All scans were conducted using a 3.0-T MRI system (Signa Premier, GE Healthcare, Waukesha, WI, USA) [15]. MRE acquisition was performed in the supine position (Fig. 1b) with a commercial pneumatic active driver (Resoundant Inc., Rochester, MN, USA) generating 30 Hz mechanical vibrations. Vibrations were transmitted to the low back using a customized belt driver at L4–L5 (Fig. 1c–e). A knee bolster was placed beneath the participants’ knees to maintain consistent lumbar positioning and minimize passive tissue strain. The belt driver was aligned symmetrically at L4–L5, with positioning verified using MRI localization images and MRI-compatible fiducial markers at the belt center [15].

The full 3D displacement vector field was acquired using a dual-saturation-dual-sensitivity spin-echo echo-planar-imaging sequence [29] with echo time/repetition time = 46/4,800 ms, six motion-encoding directions (± *x*/*y*/*z*), four phase offsets, an acquired matrix of 128 × 96 and a reconstructed matrix of 256 × 256, field of view of 38.4 × 28.8 cm^2^, and slice thickness of 4 mm with 36 axial slices. Additional anatomical MRI sequences (axial T1-weighted, axial T2-weighted fat-suppressed, sagittal T1-weighted, and sagittal T2-weighted fat-suppressed) were acquired to assess spinal and lower back musculoskeletal abnormalities.

To assess within-day and between-day SII repeatability, a subset of HV underwent three scanning sessions. Two scans (Exam 1 and Exam 2) were performed on the same day (Day 1), separated by a brief break during which participants exited the scanner and the driver belt was removed and repositioned to reset. The third scan (Exam 3) was performed approximately two weeks later (Day 2).

### SII image post-processing

MRE phase images were processed using a previously described SII pipeline to generate Dnorm [15]. The displacement field was reconstructed from the acquired MRE phase images by extracting the fundamental tissue motion component using Fourier-based analysis, from which Dnorm maps were derived (Fig. 1g). Dnorm quantifies relative motion across myofascial interfaces during MRE-induced vibration by capturing displacement discontinuities based on normalized spatial gradients of the 3D displacement field. Conceptually, Dnorm represents a relative mobility metric, with higher Dnorm values appearing as continuous bright high-intensity lines along interfaces (*e.g.*, QL–ES and ES–M in Fig. 1g), indicating greater relative slip between adjacent tissue layers and increased myofascial mobility. In contrast, lower Dnorm values, characterized by the absence of distinct high-intensity boundaries and diffuse low-intensity patterns, indicate similar displacements across the interface and reduced interfascial motion or restricted tissue mobility. Following generation of the Dnorm maps, the bilateral QL, ES, and M muscles were manually segmented to delineate inter-muscular interfaces. The QL–ES and ES–M at L3 and L4 were predefined as regions-of-interest based on their clinical relevance in CLBP. This level was selected to standardize field-of-view placement at the mid-lumbar spine (L3–L4), ensuring consistent visualization of the lower lumbar functional region influenced by MRE excitation from the L4–L5 driver belt and improving measurement reproducibility. Dnorm values were sampled across the QL–ES and ES–M and analyzed for localized intensity peaks. A unitless mobility index was computed as the mean value across each interface, summarizing spatial patterns into a single metric for statistical analysis. Higher values indicate greater interfascial mobility, as described in Supplementary Fig. 1 [15]. To assess segmentation-related reproducibility, ES and M muscles from 40 datasets (20 patients and 20 matched HVs) were independently segmented by a second rater, and inter-rater reproducibility of the mobility indices was evaluated.

### Statistical analysis

Participant demographics (age/sex/BMI) and PEG, pain duration, PCQ, PROMIS-6a, PROMIS-6b, and WPI were summarized as mean ± standard deviation. Demographics were compared between CLBP-MP and matched HV using independent-samples *t*-tests for age and BMI and chi-square or Fisher’s exact tests for sex.

For the test–retest repeatability, within-subject coefficient of variation (wCV) was calculated for each participant and inter-muscular interface as the standard deviation divided by the mean of repeated measurements, expressed as a percentage [30]. Mean wCVs with 95% confidence intervals were reported for each interface, with wCV < 10% considered excellent repeatability [30]. Bland–Altman analysis was performed to assess measurement agreement, reporting bias and limits of agreement (LoA) [31, 32]. Inter-rater reproducibility of mobility indices was assessed using wCV and Bland–Altman analysis.

To characterize the myofascial mobility in HV, the effects of laterality (left *versus* right), interface (QL–ES *versus* ES–M), and vertebral level (L3 *versus* L4) on the mobility index were evaluated using repeated-measures ANOVA. Normality was evaluated using the Lilliefors test, confirming that all distributions did not significantly deviate from normality. Significant effects were examined using Tukey’s Honestly Significant Difference (HSD) post hoc test with Benjamini–Hochberg False Discovery Rate (BH-FDR) correction, yielding *q* values. In HVs, unadjusted associations between mobility indices and age/sex/BMI were assessed using Pearson correlation and multivariable linear regression as a sensitivity analysis.

To assess myofascial mobility in patients with CLBP-MP, mobility indices at the QL–ES and ES–M at the L3 and L4 were compared across three pain severity groups defined by PEG score, with exploratory subgroup analyses stratified by FM status, using one-way ANOVA. Group differences were further examined using Tukey’s HSD post hoc tests with BH-FDR correction, and mean differences with 95% confidence intervals and standardized effect sizes (Cohen’s *d*) were computed.

Within the patient cohort, Pearson correlation analysis was performed to assess associations between mobility indices and clinical measures (PEG, pain duration, PCQ, PROMIS-6a, PROMIS-6b, and WPI), both in the full patient cohort and separately within FM and non-FM subgroups.

The significance level was set at *p* < 0.05. All statistical analyses were performed using MATLAB R2023a.

## Results

### Participant demographics and clinical characteristics

Demographic and clinical characteristics are summarized in Table 1a. Group comparisons showed no significant differences in age/sex/BMI, indicating appropriate matching (*p* ≥ 0.893). Patients reported moderate pain severity (PEG: 5.47 ± 2.36) [23, 28], with a highly variable pain duration (85.3 ± 96.1 months). Pain catastrophe, sleep disturbance, and physical function were also moderate, with mean scores of 18.75 ± 12.18 (PCQ, 0–52), 18.74 ± 6.36 (PROMIS-6a, 0–30), and 18.95 ± 5.64 (PROMIS-6b, 0–30), respectively [33, 34]. Among patients with comorbid FM, the mean WPI score was 11.55 ± 3.83 (range 0–19) (Table 1b).

**Table 1:** Demographic and clinical characteristics of the studied subjects

Characteristic	All HV (*n* = 41)	HV repeatability subset (*n* = 18)	CLBP-MP (*n* = 20)	Matched HV (*n* = 20)		*p* value^*^
a						
Sex, *n*	Male: 16 Female: 25	Male: 9 Female: 9	Male: 2 Female: 18	Male: 2 Female: 18		1.000
Age (years)	39.10 ± 11.82	37.83 ± 11.29	43.45 ± 13.57	43.25 ± 12.79		0.962
BMI (kg/m²)	25.35 ± 4.31	24.91 ± 2.69	24.56 ± 3.23	24.43 ± 3.24		0.893
b						
PEG (*n* = 20)					5.47 ± 2.36	
Pain duration (months) (*n* = 20)					85.30 ± 96.14	
PCQ (*n* = 20)					18.75 ± 12.18	
PROMIS-6a sleep disturbance (*n* = 19)					18.74 ± 6.36	
PROMIS-6b physical function (*n* = 19)					18.95 ± 5.64	
Widespread pain index (*n* = 11)					11.55 ± 3.83	

a: Demographic characteristics, including age, sex, and BMI, are shown for patients with CLBP-MP, age-, sex-, and BMI-matched HV, the full healthy volunteer cohort, and the healthy volunteer repeatability subset

b: Clinical characteristics for patients with CLBP-MP, including PEG score, pain duration, PCQ score, PROMIS-6a sleep disturbance score, PROMIS-6b physical function score, and widespread pain index score. Data are reported as mean ± standard deviation. *p* values reflect comparisons between patients with CLBP-MP and matched HV

*n* Number of participants included in each analysis, *BMI* Body mass index, *CLBP-MP* Chronic low back pain with myofascial pain features, *HV* Healthy volunteers, *PROMIS-6a* Patient-reported outcomes measurement information system

### Test–retest repeatability of SII measurement

**Fig. 2:**
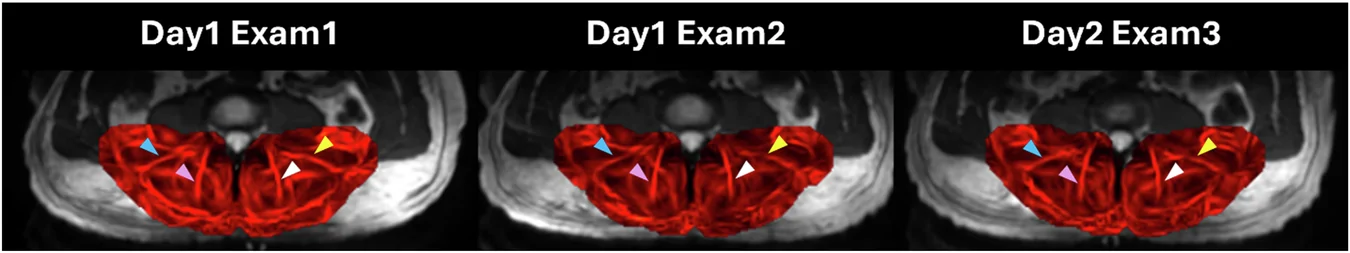
Example of Dnorm maps overlaid on the corresponding MRE magnitude images from two repeated exams (Exam 1 and Exam 2) on Day 1 and the third exam (Exam 3) on Day 2. In all three scans, bright, high-intensity Dnorm values are visible along the QL–ES and ES–M interfaces, illustrating consistent interface contrast across sessions. Blue, pink, white, and yellow arrows indicate right QL–ES, right ES–M, left ES–M, and left QL–ES interfaces, respectively

**Fig. 3:**
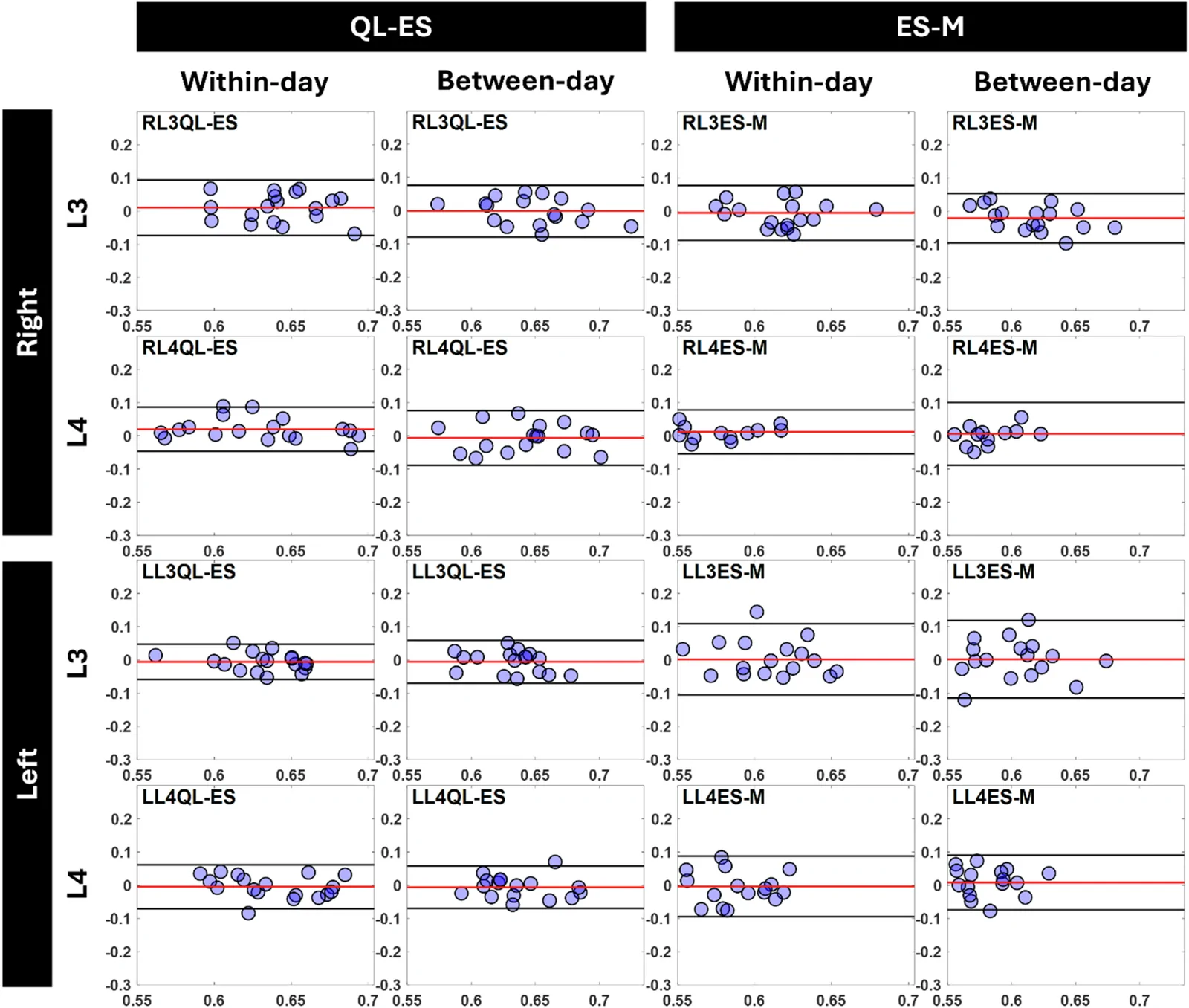
Bland–Altman plots for within-day (1 and 3 columns) and between-day (2 and 4 columns) repeatability. Blue dots represent the differences between the two exams of the Dnorm mobility index on the vertical axis, while the mean of those measurements is plotted on the horizontal axis. Red and black lines indicate the mean difference (bias) and the upper and lower LoA, respectively. ES, Erector spinae; L, Left; M, Multifidus; QL, Quadratus lumborum; R, Right

Representative Dnorm from repeated same-day (Exams 1 and 2) and separate-day (Exam 3) scans are shown in Fig. 2. Within-day and between-day repeatability at bilateral QL–ES and ES–M at L3 and L4 are summarized (Table 2) and visualized using Bland–Altman plots (Fig. 3).

**Table 2:** Repeatability of the Dnorm mobility index

			Within-day (*n* = 18)			Between-day (*n* = 17)		
			Mean wCV	Bias	LoA	Mean wCV	Bias	LoA
L	ES–M	L3	5.14 (3.43, 6.85)	0.002	(-0.106, 0.109)	5.26 (3.11, 7.41)	0.002	(-0.115, 0.119)
		L4	4.59 (3.12, 6.06)	-0.004	(-0.095, 0.088)	4.28 (2.91, 5.64)	0.008	(-0.075, 0.090)
	QL–ES	L3	2.40 (1.51, 3.29)	-0.006	(-0.059, 0.048)	2.99 (1.99, 3.99)	-0.006	(-0.070, 0.059)
		L4	3.08 (2.10, 4.07)	-0.005	(-0.071, 0.062)	2.82 (1.77, 3.86)	-0.006	(-0.070, 0.058)
R	ES–M	L3	4.13 (2.92, 5.33)	-0.006	(-0.089, 0.077)	3.99 (2.67, 5.32)	-0.022	(-0.096, 0.053)
		L4	3.63 (2.25, 5.01)	0.012	(-0.054, 0.078)	4.24 (2.01, 6.46)	0.006	(-0.089, 0.101)
	QL–ES	L3	4.13 (3.08, 5.19)	0.010	(-0.074, 0.094)	3.74 (2.80, 4.69)	-0.002	(-0.080, 0.077)
		L4	3.11 (1.64, 4.59)	0.020	(-0.046, 0.087)	3.77 (2.46, 5.09)	-0.006	(-0.089, 0.076)

Mean wCV (%), bias, and LoA for within-day and between-day repeatability of the Dnorm mobility index at the right and left L3 and L4 QL–ES and ES–M interfaces. Values in parentheses indicate the 95% confidence intervals for wCV and the lower and upper bounds of the LoA

*ES* Erector spinae, *L* Left, *LoA* Limits of agreement, *M* Multifidus, *QL* Quadratus lumborum, *R* Right, *wCV* Within-subject coefficient of variation

Mobility indices demonstrated excellent repeatability across all interfaces, sides, and vertebral levels, with mean wCV values below 10%. Within-day bias ranged from -0.006 to 0.020 and between-day bias from -0.022 to 0.008, indicating negligible bias. Between-day measurements were similarly reproducible, with slightly wider LoA for all interfaces except QL–ES and ES–M on the right L3 and left L4, indicating minimal day-to-day measurement variability. No consistent directional bias was observed across sides, interfaces, or vertebral levels. Mobility indices from all three exams are shown in Supplementary Fig. 2. Inter-rater reproducibility demonstrated excellent agreement, with wCV below 5% for both individual-side and side-averaged ES–M mobility indices at L3 and L4 (Supplementary Table S1). Bland–Altman analysis showed minimal bias and acceptable LoA between raters (Supplementary Fig. 3).

### HV mobility index and correlations with age, sex, and BMI

**Fig. 4:**
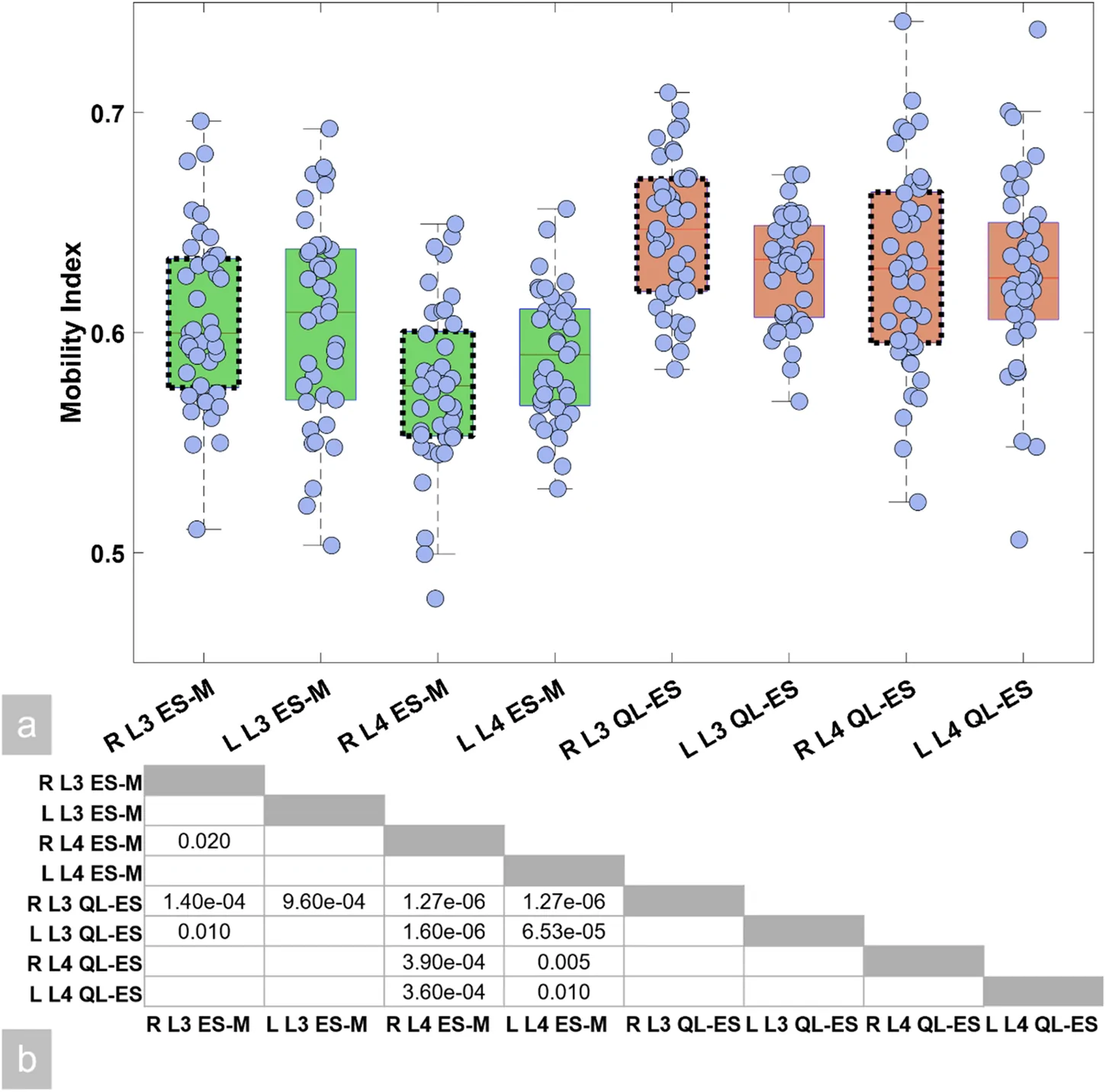
**a** Mobility index box plots for HV (*n* = 41), comparing right and left sides for ES–M (green) and the QL (orange) at L3 and L4. Blue dots represent individual values, and black dotted outlines indicate the right side. **b** Statistical significance of *q* values from repeated-measures ANOVA with Tukey’s HSD *post hoc* test with Benjamini–Hochberg false discovery rate correction across the eight interfaces. ES, Erector spinae; L, Left; M, Multifidus; QL, Quadratus lumborum; R, Right

As shown in Fig. 4, repeated-measures ANOVA revealed significant effects of interface (F_1,40_ = 58.34, *p* < 0.001) and vertebral level (F_1,40_ = 11.87, *p* = 0.001), as well as significant laterality × level interaction (F_1,40_ = 5.21, *p* = 0.028). No significant effect of laterality (F_1,40_ = 0.16, *p* = 0.690) or other interactions were observed. Post hoc Tukey HSD comparisons with BH-FDR correction identified significant interface- and level-dependent differences, indicating normal anatomical variability in myofascial mobility, with generally greater at the QL–ES than the ES–M. No significant vertebral level differences were observed for QL–ES, whereas at the right ES–M was significantly greater at L3 than L4.

Mobility indices were averaged across sides and analyzed for associations with age/sex/BMI in HV. No significant unadjusted associations with age/sex/BMI were observed at any interface or vertebral level (Supplementary Fig. 4). Multivariable linear regression similarly showed no consistent associations, apart from a small association at the L3 QL–ES (*p* = 0.040-0.045 for BMI and sex; Supplementary Table S2).

### Myofascial mobility and clinical correlations in CLBP-MP patients

**Fig. 5:**
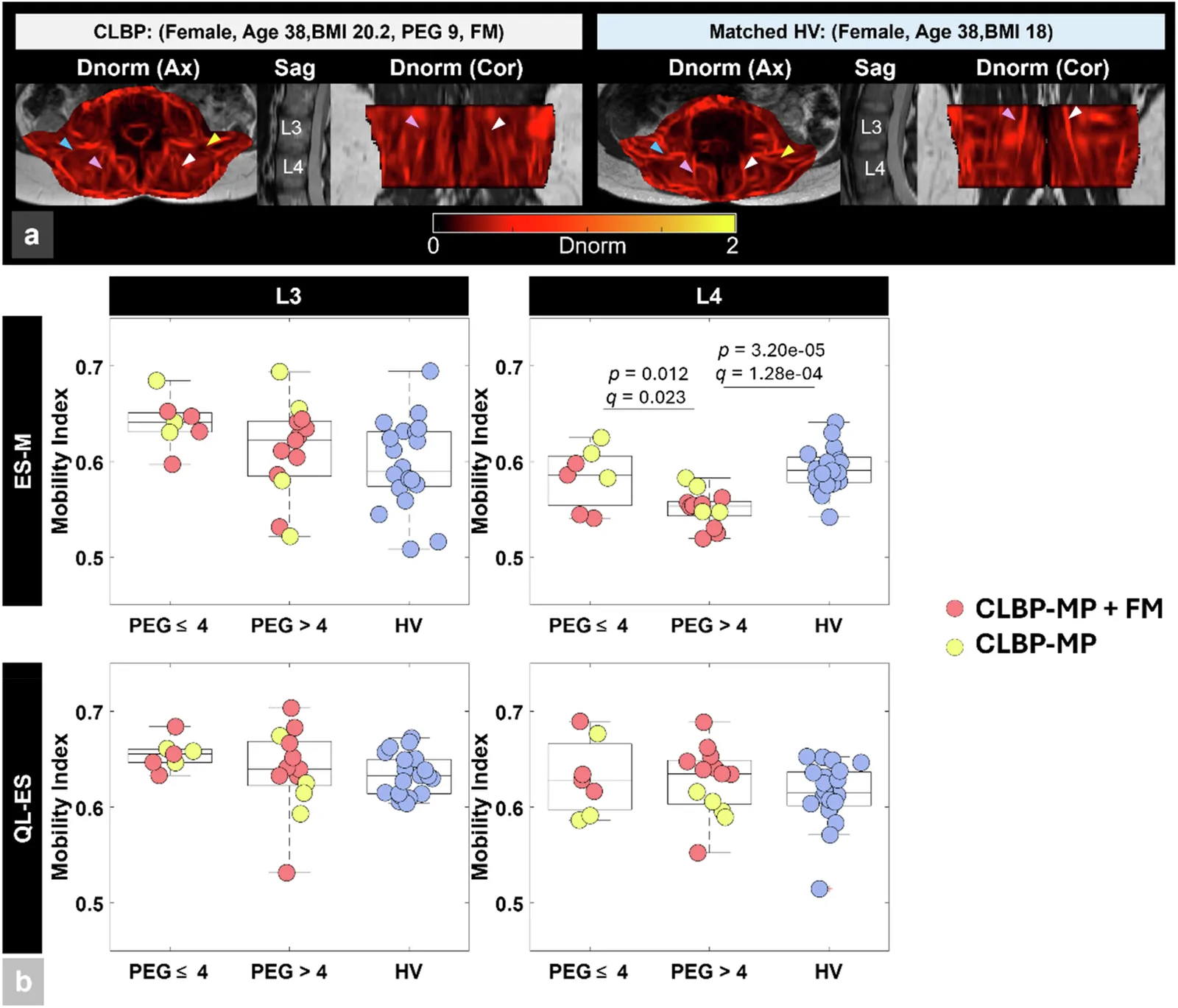
**a** Representative comparison of Dnorm maps between a patient with CLBP-MP (columns 1–3) and a matched healthy volunteer (columns 4–6). Images show axial Dnorm at L3–L4, sagittal T2-weighted, and coronal Dnorm at the same level. Blue, pink, white, and yellow arrows indicate right QL–ES, right ES–M, left ES–M, and left QL–ES interfaces, respectively. Axial and coronal Dnorm maps are overlaid on T2-weighted images. Compared with the well-defined, traceable myofascial interface observed in the matched healthy volunteer, the patient showed decreased Dnorm contrast and discontinuity at the ES–M interface, suggesting restricted interfacial mobility. **b** Box plots of mobility indices at the L3 and L4 ES–M and QL–ES interfaces across three groups: patients with mild pain (PEG ≤ 4), patients with moderate-to-severe pain (PEG > 4), and HV (blue symbols). Red and yellow symbols denote patients with and without comorbid FM, respectively. Statistical significance is indicated by corresponding *p* and *q* values. Ax, Axial; BMI, Body mass index; CLBP-MP, Chronic low back pain with myofascial pain features; Cor, Coronal; Dnorm, Maximal normalized displacement discontinuity; ES, Erector spinae; FM, Fibromyalgia; HV, Healthy volunteers; M, Multifidus; PEG, Pain, enjoyment and general activity; QL, Quadratus lumborum; Sag, Sagittal

Representative Dnorm maps from a CLBP-MP and an age/sex/BMI-matched HV demonstrated reduced ES–M mobility in the patient (Fig. 5a). Compared with the matched HV, the patient showed decreased Dnorm contrast and discontinuity at the L4 ES–M, suggesting restricted interfascial mobility.

Because pain was not consistently unilateral and no laterality-dependent effects were observed in the HV, left and right mobility indices were averaged for each interface. One-way ANOVA demonstrated a significant group effect for the L4 ES–M mobility index (F(2,37) = 13.23, *p* = 4.64e-05). Post hoc Tukey HSD tests with BH-FDR correction revealed that patients with moderate-to-severe pain (PEG > 4) exhibited significantly reduced L4 ES–M mobility compared with patients with mild pain (PEG ≤ 4; *p* = 0.012, *q* = 0.023) and HV (*p* = 3.20e-05, *q* = 1.28e-04), with large effect sizes (Table 3). No other group differences in mobility indices were observed across the remaining interfaces or vertebral levels (Fig. 5b). Consistent with the primary analysis, results at the L4 ES–M remained quantitatively similar when using the second rater’s segmentations (PEG > 4 *versus* HV: *p* = 0.009, *q* = 0.007). Accordingly, all subsequent analyses were based on the first rater’s segmentations. In exploratory analyses stratified by FM status, CLBP-MP with FM also showed significantly reduced L4 ES–M mobility compared with HV (F(2,37) = 11.57, *p* = 1.25e-04; post hoc Tukey HSD: *p* = 7.91e-05, *q* = 3.16e-04; Supplementary Fig. S5). No other significant subgroup differences were observed.

**Table 3:** Differences in bilateral ES–M and QL–ES mobility indices

	Mean difference (95% CI)	*p* value	*q* value	Cohens’*d*
ES–M L3 (PEG > 4 *versus* PEG < = 4)	-0.029 (-0.079, 0.022)	0.352	n/a	0.686
ES–M L3 (PEG > 4 *versus* HV)	0.014 (-0.024, 0.053)	0.631	n/a	0.309
ES–M L3 (PEG < = 4 *versus* HV)	0.043 (-0.004, 0.091)	0.078	0.104	1.031
ES–M L4 (PEG > 4 *versus* PEG < = 4)	-0.033 (-0.059, -0.006)	0.012	0.023	1.405
ES–M L4 (PEG > 4 *versus* HV)	-0.042 (-0.062, -0.022)	3.20e-05	1.28e-04	1.986
ES–M L4 (PEG < = 4 *versus* HV)	-0.009 (-0.033, 0.016)	0.661	n/a	0.352
QL–ES L3 (PEG > 4 *versus* PEG < = 4)	-0.017 (-0.052, 0.017)	0.437	n/a	0.473
QL–ES L3 (PEG > 4 *versus* HV)	0.003 (-0.022, 0.029)	0.943	n/a	0.109
QL–ES L3 (PEG < = 4 *versus* HV)	0.021 (-0.011, 0.053)	0.262	0.262	1.030
QL–ES L4 (PEG > 4 *versus* PEG < = 4)	-0.004 (-0.044, 0.036)	0.965	n/a	0.113
QL–ES L4 (PEG > 4 *versus* HV)	0.014 (-0.017, 0.044)	0.524	n/a	0.401
QL–ES L4 (PEG < = 4 *versus* HV)	0.018 (-0.020, 0.055)	0.484	n/a	0.515

Mean differences in bilateral ES–M and QL–ES mobility indices at L3 and L4 across pain severity groups and HV. Values are reported as mean difference (95% confidence intervals). *p* values are unadjusted; *q* values are Benjamini–Hochberg false discovery rate-corrected where applicable. Cohen’s *d* denotes the standardized absolute effect size. Large effect sizes in the absence of statistical significance likely reflect small subgroup sample sizes and should be interpreted cautiously when confidence intervals include zero

*CI* Confidence interval, *ES–M* Erector spinae-multifidus, *HV* Healthy volunteers, *PEG* Pain, enjoyment, and general activity, *QL–ES* Quadratus lumborum–erector spinae.

**Fig. 6:**
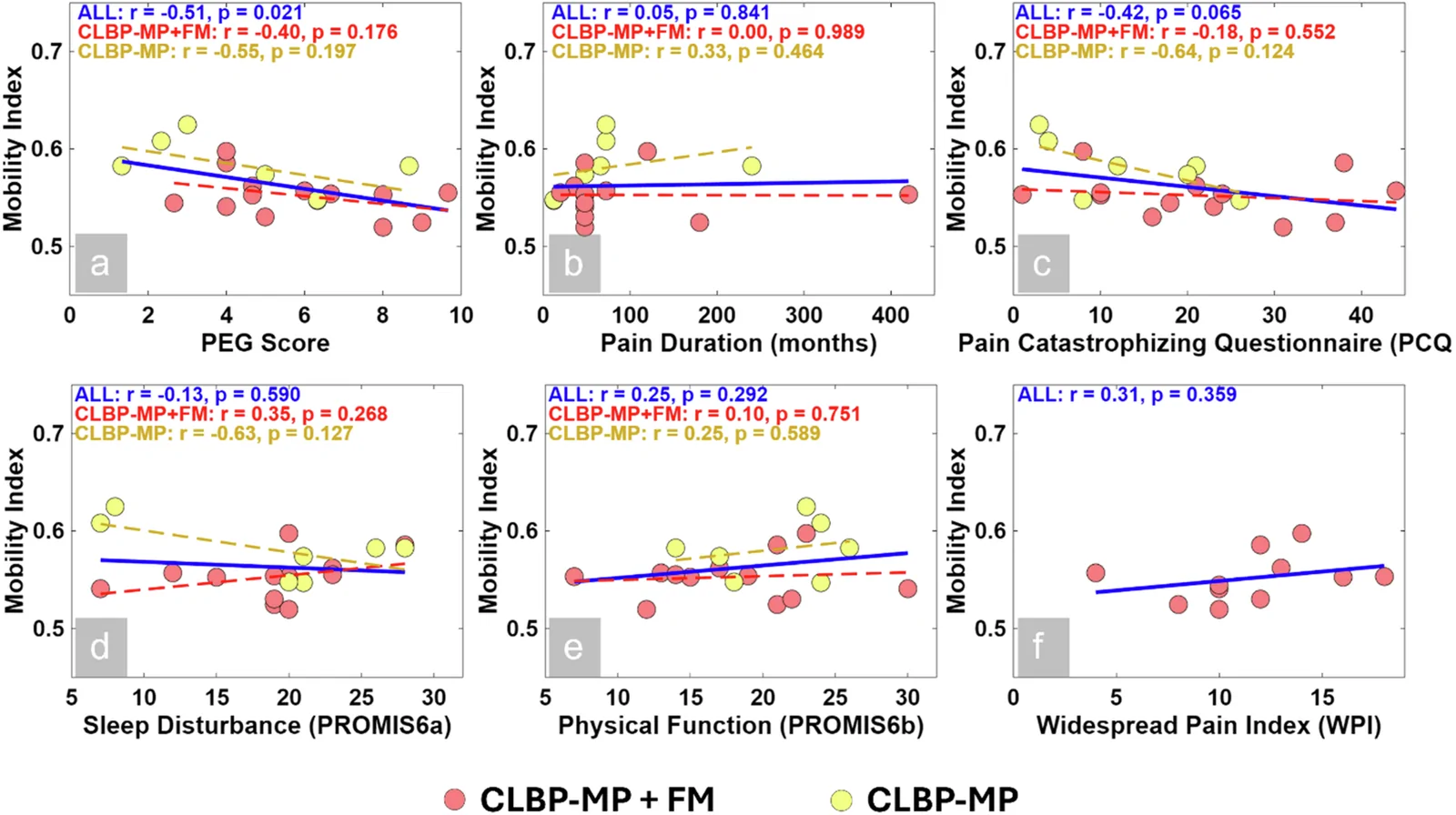
Correlations between the mobility index at the L4 ES–M interface (vertical axis) and clinical measures (horizontal axis): PEG score (**a**), pain duration (**b**), PCQ score (**c**), sleep disturbance (**d**), physical function (**e**), and widespread pain index (**f**), in patients with CLBP with myofascial pain features. Regression fits are shown for all patients (blue) and for patients with (red) and without (yellow) FM. Pearson correlation coefficients (*r*) and corresponding *p* values are shown in each panel. Red and yellow symbols denote individual patients with and without comorbid FM, respectively. CLBP-MP, Chronic low back pain with myofascial pain features; FM, Fibromyalgia; PCQ, Pain catastrophizing questionnaire; PEG Pain, enjoyment, and general activity; PROMIS-6a, Patient-reported outcomes measurement information system sleep disturbance short form; PROMIS-6b, Patient-reported outcomes measurement information system physical function short form; WPI, Widespread pain index

Within the patient cohort, L4 ES–M mobility showed a moderate negative correlation with PEG score (*r* = −0.51, *p* = 0.021; Fig. 6), whereas PEG score was not significantly associated with mobility at other interfaces. When stratified by FM status, subgroup analyses did not demonstrate consistent significant associations. Mobility indices showed no significant correlations with pain duration, PCQ, PROMIS-6a, PROMIS-6b, or WPI across most interfaces or vertebral levels. A few nominally significant correlations were observed, including associations between PEG score and L3 QL–ES mobility index in the CLBP-MP subgroup, pain duration and L3 ES–M mobility in the CLBP-MP + FM subgroup, and sleep disturbance and L3 QL–ES mobility in the CLBP-MP + FM subgroup, as well as the pooled patient cohort (Supplementary Fig. S6).

Given the limited subgroup sample sizes and the lack of consistency across regions and measures, these findings are considered exploratory and should be interpreted cautiously.

## Discussion

This study evaluated the clinical feasibility of the MRE-based SII biomarker, Dnorm, for quantifying inter-muscular myofascial mobility at the QL–ES and ES–M at L3 and L4. Three complementary investigations assessed test–retest repeatability, characterization of inter-muscular mobility in HV and its associations with demographic factors, and compared mobility according to pain severity in patients with CLBP-MP while examining associations with self-reported clinical measures. Overall, Dnorm provided highly repeatable and anatomically sensitive measurements of myofascial mobility. A significant reduction in mobility was observed at the L4 ES–M in patients with higher pain severity, and this finding was associated with PEG score, while other examined interfaces showed no consistent group differences.

The Dnorm mobility index demonstrated excellent test–retest repeatability across all lumbar levels and interfaces, with variability well below the predefined 10% threshold [35], supporting its robustness as a quantitative biomarker. Both within-day and between-day measurements showed minimal variability and strong agreement, indicating that Dnorm is temporally stable and insensitive to short-term physiological or experimental fluctuations. The narrow LoA provides a reference for distinguishing measurement error from true biological change, suggesting that changes exceeding these ranges may represent clinically meaningful longitudinal differences [31, 32]. Slightly greater variability was observed at L3 compared with L4, particularly at the ES–M. This may reflect anatomical and biomechanical factors, including increased soft tissue thickness [36–38], greater inter-muscular boundary complexity [39, 40], and inter-individual differences in lumbar curvature [41, 42]. However, this pattern was not uniform across all comparisons, suggesting that local factors such as postural variation, asymmetric muscle activation, or subtle differences in driver positioning may also contribute. Overall, the high repeatability of the Dnorm mobility index and these reference ranges support its use for longitudinal monitoring and for detecting clinically significant changes in lumbar muscle mobility in CLBP-MP.

In HV, myofascial mobility was bilaterally symmetric and varied across anatomical interfaces and vertebral levels, consistent with normal regional variability. Mobility indices showed no meaningful associations with age/sex/BMI in either unadjusted or multivariable analyses, suggesting that demographic factors explain little of the variability in lumbar inter-muscular mobility. Although *ex vivo* studies have reported age- and hormone-related changes in fascial composition and hydration, including increases in collagen crosslinking [43], reduced elastin and hyaluronan turnover [43, 44], and estrogen-dependent regulation of fascial hydration [45, 46], their impact on *in vivo* inter-muscular sliding remains uncertain and may depend on loading conditions, posture, and neuromuscular activation. Importantly, the magnitude and site-specificity of the mobility reduction observed in CLBP-MP patients suggest that pain-related pathological processes may outweigh normal biological variability.

CLBP-MP showed reduced myofascial mobility at the L4 ES–M, with larger reductions observed in those with higher pain severity. This reduction correlated moderately with PEG scores, suggesting an association between segment-specific inter‑muscular mobility and pain intensity and/or functional interference. The observed regional specificity may reflect biomechanical vulnerability, as the L4–L5 segment bears high mechanical loads [37] and contributes substantially to lumbar stability [47] and force transmission [48]. The M and ES muscles have complementary roles in segmental stabilization and torque generation, respectively [49, 50]; impaired inter-muscular glide may alter local mechanical coupling and movement patterns, potentially contributing to activity-related pain [51–53]. Reduced Dnorm values may arise from a combination of mechanisms, including altered neuromuscular activation [4], pain-related movement adaptations [54], and changes in inter-muscular tissue properties [55]. In patients with comorbid FM, reduced mobility may additionally reflect contributions from central sensitization-related processes, such as increased baseline muscle tone [56], sustained muscle co-contraction [56, 57], and impaired microvascular flow [58]. Notably, L4 ES–M mobility correlated with PEG score but not with pain duration, pain catastrophizing, sleep disturbance, physical function, or WPI. Because PEG captures pain intensity and activity interference [23], it may be more closely related to movement-associated mechanical factors [59, 60], whereas pain duration and psychosocial measures may reflect different dimensions of the pain experience, including cumulative exposure and central pain modulation [61]. These findings suggest that impaired ES–M mobility may represent a localized mechanical correlation of pain severity rather than a nonspecific marker of global symptom burden.

FM status was common in this cohort, and patients with comorbid FM tended to have higher PEG scores and lower L4 ES–M mobility, warranting further evaluation in larger, well-powered studies. Given the known overlap between FM and localized myofascial pain [62], these findings do not exclude central sensitization, but rather suggest that focal mechanical mobility alterations at the L4 ES–M may coexist with broader pain modulation processes. In contrast, no consistent differences were observed at other inter-muscular interfaces or vertebral levels, supporting region-specific alterations rather than global lumbar myofascial dysfunction. Accordingly, impaired L4 ES–M mobility may represent a focal mechanical correlate of pain severity rather than a generalized reduction in lumbar myofascial mobility, although limited subgroup sizes and muscle-specific heterogeneity may also have constrained detection of more subtle effects. These interpretations are hypothesis-generating, as the cross-sectional design precludes determination of causality.

These findings align with an emerging understanding of the functional role of myofascial tissue as a dynamic, mechanosensitive network involved in force transmission, proprioception, and nociceptive modulation. Dysregulation of interlayer sliding through immobility, repetitive strain, or inflammation has been associated with localized stiffening, extracellular matrix remodeling, and reduced inter-muscular motion [63–66]. In this context, the reduced mobility observed in CLBP-MP may reflect localized myofascial dysfunction. Noninvasive imaging approaches, including ultrasound shear wave elastography and MRE-based SII, have begun to quantify these mechanical abnormalities *in vivo* [7, 15, 67]. While ultrasound can detect impaired fascial gliding, it may be limited by tissue depth, acoustic window, and operator variability. In contrast, MRE-based SII offers a larger field-of-view and objective, segment-specific measures of inter-muscular mobility. By demonstrating high repeatability and anatomically specific associations with pain severity, this study supports SII as an early-stage quantitative biomarker candidate for mechanobiology-informed phenotyping and longitudinal assessment in CLBP.

Several limitations should be acknowledged. First, repeatability was assessed only in HV with BMI ≤ 30. Therefore, additional reproducibility studies in larger patient cohorts with more diverse characteristics, including higher BMI, are needed to establish robustness across broader clinical settings. Although manual segmentation demonstrated excellent inter-rater reproducibility, the current workflow still requires trained observers and has not yet been fully automated. Development and validation of automated or semi-automated segmentation approaches will be important for improving scalability, reducing operator workload, and facilitating clinical translation. Second, the cross-sectional, single-center design limits causal inference regarding whether reduced myofascial mobility contributes to pain severity or reflects a secondary adaptation to chronic pain, and the findings have not yet been externally validated. Third, subgroup sizes, especially for patients with mild pain and for FM-stratified analyses, were modest, limiting statistical power and increasing uncertainty around subgroup-specific findings. Consequently, these analyses should be regarded as descriptive and exploratory rather than confirmatory. Fourth, minor imbalances in age/sex/BMI across groups may have introduced residual confounding, suggesting the need for larger, more balanced cohorts. Fifth, inclusion of patients with comorbid FM introduced clinically relevant phenotype heterogeneity (*i.e.*, variation in FM status and pain severity across patients) and complicates mechanistic attribution of reduced mobility findings to localized lumbar myofascial dysfunction *versus* broader central sensitization or mixed pain mechanisms. Notably, FM-stratified analyses did not reveal consistent mobility reductions beyond the focal L4 ES–M, suggesting relative regional specificity; however, larger, well-phenotyped cohorts are required to disentangle peripheral mechanical contributors from centralized pain processes. Dnorm should be interpreted as an imaging-based indicator of relative inter-muscular mobility differences rather than a standalone diagnostic criterion. Although reduced mobility was associated with greater pain severity, clinically meaningful cutoffs and diagnostic thresholds remain undefined. Finally, this single-center study lacked external validation, which may limit generalizability and raise the possibility of center-specific effects. Larger multicenter, longitudinal studies are needed to validate the biomarker, establish normative reference ranges and clinically meaningful thresholds, and determine its utility for clinical decision-making.

Despite these limitations, the findings support the feasibility of MRE-based SII for quantifying localized myofascial mobility impairments associated with pain severity and provide a foundation for future validation studies to establish its clinical utility and evaluate myofascial or force-based interventions.

In summary, MRE-based SII provided highly repeatable and anatomically sensitive measures of lumbar inter-muscular mobility. Reduced L4 ES–M mobility in CLBP-MP and its association with greater pain severity support the feasibility of SII as an early-stage quantitative imaging biomarker candidate.

## Supplementary information

**Additional file:** **Table S1:** Mean within-subject coefficient of variation (%), bias, and limits of agreement for inter-rater reproducibility of the Dnorm mobility index at the right and left L3 and L4 erector spinae-multifidus interfaces and their bilateral average. Values in parentheses indicate the 95% confidence intervals for within-subject coefficient of variation and for the lower and upper limits of agreement. **Table S2:** Multivariable linear regression was performed for each lumbar interface (erector spinae-multifidus and quadratus lumborum-erector spinae at L3 and L4). The estimate (β) represents the regression coefficient for each predictor (Age, BMI, Sex), p-value indicates statistical significance. **Figure S1:** Mobility index calculation workflow **a.** Manual segmentation of the quadratus lumborum, erector spinae, and multifidus muscles to delineate inter-muscular interfaces (white arrows). **b.** Sampling of Dnorm values along interface-orthogonal line profiles (white lines) at the segmented boundaries (colored). For each interface, sampled Dnorm profiles were classified as peak-type or non-peak-type based on the presence of a localized intensity maximum along the profile. Peak-type profiles contributed normalized values, whereas non-peak-type profiles were assigned a value of zero. The mobility index was defined as the mean of all profile values sampled along the interface, yielding a single, unitless measure of relative inter-muscular mobility. **Figure S2:** Boxplot of the Dnorm mobility index from three exams for all subjects (n=18) at the quadratus lumborum-erector spinae and erector spinae-multifidus interfaces, at L3 and L4 levels, on the left (the first and third columns) and right (the second and fourth columns) side respectively. The red dotted lines indicate the mean values of the Dnorm mobility index across all subjects. Note: Data from Exam 3 for subject 9 was excluded, so the results reflect only Exams 1 and 2. **Figure S3:** Bland-Altman plots for inter-rater reproducibility of the Dnorm mobility index at the L3 (top row) and L4 (bottom row) erector spinae-multifidus interfaces for the right, left, and bilateral average. Blue dots represent the differences between the two raters (R2 - R1), plotted against the mean of the two raters. The red solid line indicates the mean difference (bias), and the black dashed lines indicate the upper and lower limits of agreement. **Figure S4:** Correlations between mobility index (vertical axis) and Age (a), Sex (**b**), and BMI (**c**) (horizontal axes) in HV (n=41) at L3 and L4 for the quadratus lumborumerector spinae and erector spinae-multifidus. White and blue dots indicate male and female individuals, respectively. Blue lines show linear regression fits. Pearson’s r and p-values are reported in each panel. **Figure S5:** Box plots of mobility indices at the L3 and L4 erector spinae-multifidus and quadratus lumborum-erector spinae interfaces across three groups: chronic low back pain patients with myofascial pain features without or with fibromyalgia and healthy volunteers (blue symbols). Green and grey symbols denote patients with moderate-to-severe pain (PEG > 4) and with mild pain (PEG ≤ 4), respectively. Statistical significance is indicated by corresponding p and q values. **Figure S6:** Correlations between mobility index (vertical axis) and six clinical measures (horizontal axis): PEG score, pain duration, Pain Catastrophizing Questionnaire, Sleep Disturbance, Physical Function, and Widespread Pain Index. Columns 1-6 show these measures across patients for L3 erector spinae-multifidus, L3 and L4 quadratus lumborum-erector spinae (rows 1-3). Regression fits are shown for all patients (blue) and for patients with (red) and without (yellow) fibromyalgia. Pearson’s r and corresponding p-values are shown in each panel. Red and yellow plots represent patients with chronic low back pain with myofascial pain features, with and without FM, respectively.

## Data Availability

The datasets analysed during the current study are available from the corresponding author on reasonable request.

## Abbreviations

BH-FDR: Benjamini–Hochberg false discovery rate
BMI: Body mass index
CLBP: Chronic low back pain
CLBP-MP: CLBP with myofascial pain features
Dnorm: Maximal normalized displacement discontinuity
ES: Erector spinae
ES–M: ES–multifidus
FM: Fibromyalgia
HSD: Honestly significant difference
HV: Healthy volunteers
LoA: Limits of agreement
MRE: Magnetic resonance elastography
MRI: Magnetic resonance imaging
PCQ: Pain catastrophizing questionnaire
PEG: Pain, enjoyment, and general activity
PROMIS: Patient-reported outcomes measurement information system
QL: Quadratus lumborum
SII: Slip interface imaging
wCV: Within-subject coefficient of variation
