# Supplementary material for: Reduced lumbar myofascial mobility in chronic low back pain quantified using MR elastography-based slip interface imaging
Source: Eur Radiol Exp. 2026 Oct 1;10:148. doi: 10.1186/s41747-026-00812-0 (PMC13631199; doi:10.1186/s41747-026-00812-0)
Supplement: Supplementary file 1 [file 41747_2026_812_MOESM1_ESM.pdf]

Reduced Lumbar Myofascial Mobility in Chronic Low Back Pain Quantified Using MR Elastography-Based Slip Interface Imaging

ELECTRONIC SUPPLEMENTARY MATERIAL

Eur Radiol Exp (2026) Hojo E, Ito D, Sui Y et al.

		Mean wCV	Bias	LoA
L3	Right	4.741 (3.474, 6.008)	0.029	(-0.058, 0.116)
	Left	3.461 (2.257, 4.666)	0.016	(-0.061, 0.092)
	Right+Left (average)	3.651 (2.651, 4.651)	0.022	(-0.045, 0.090)
L4	Right	3.892 (2.968, 4.815)	-0.007	(-0.081, 0.067)
	Left	4.202 (3.254, 5.149)	-0.019	(-0.094, 0.056)
	Right+Left (average)	3.025 (2.252, 3.798)	-0.013	(-0.070, 0.044)

Table S1: Mean within-subject coefficient of variation (%), bias, and limits of agreement for inter-rater reproducibility of the Dnorm mobility index at the right and left L3 and L4 erector spinae-multifidus interfaces and their bilateral average. Values in parentheses indicate the 95% confidence intervals for within-subject coefficient of variation and for the lower and upper limits of agreement.[1]

[1] LoA, limits of agreement; wCV, within-subject coefficient of variation

Mobility outcome	Predictor	Estimate (β)	SE	tStat	*p*-value
**ES-M L3**	Age	0.000367	0.000552	0.666	0.510
	BMI	-0.000608	0.001469	-0.414	0.682
	Sex	-0.018334	0.013403	-1.368	0.180
**ES-M L4**	Age	0.000601	0.000367	1.636	0.110
	BMI	0.000178	0.000977	0.182	0.857
	Sex	0.005572	0.008917	0.625	0.536
**QL-ES L3**	Age	3.16e-05	0.000356	0.089	0.930
	BMI	-0.001959	0.000946	-2.070	0.045
	Sex	-0.018335	0.008633	-2.124	0.040
**QL-ES L4**	Age	-0.000635	0.000487	-1.304	0.200
	BMI	-0.001676	0.001295	-1.294	0.204
	Sex	-0.013593	0.011817	-1.150	0.257

Table S2: Multivariable linear regression was performed for each lumbar interface (erector spinae-multifidus and quadratus lumborum-erector spinae at L3 and L4). The estimate (β) represents the regression coefficient for each predictor (Age, BMI, Sex), *p*-value indicates statistical significance. [2]

[2] BMI, body mass index; ES, erector spinae; M, multifidus; SE, standard error of the estimate; tStat, t-statistic; QL, quadratus lumborum

Supplementary Figures

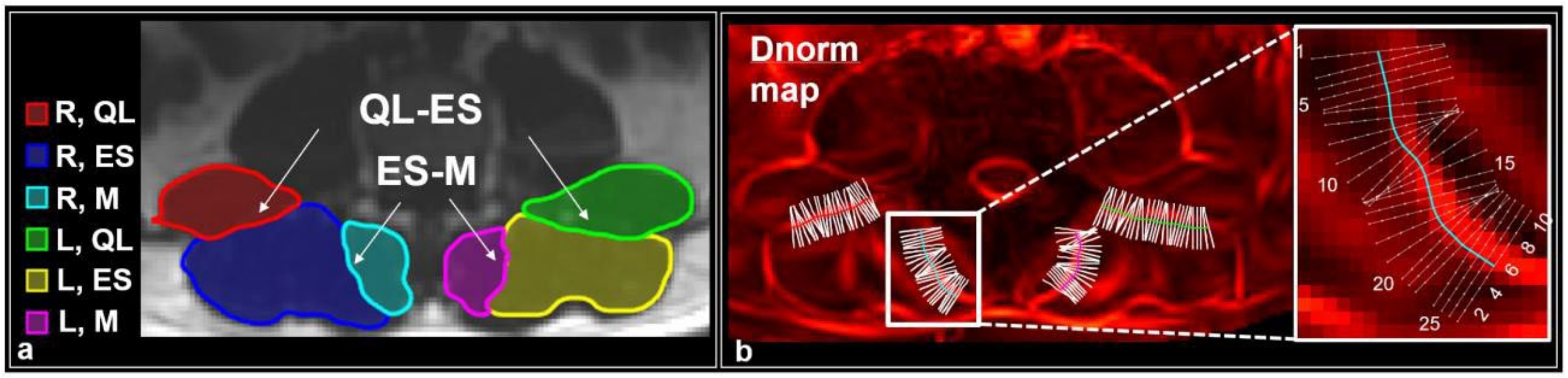

S1: Mobility index calculation workflow a. Manual segmentation of the quadratus lumborum, erector spinae, and multifidus muscles to delineate inter-muscular interfaces (white arrows).

b. Sampling of Dnorm values along interface-orthogonal line profiles (white lines) at the segmented boundaries (colored). For each interface, sampled Dnorm profiles were classified as peak-type or non-peak-type based on the presence of a localized intensity maximum along the profile. Peak-type profiles contributed normalized values, whereas non-peak-type profiles were assigned a value of zero. The mobility index was defined as the mean of all profile values sampled along the interface, yielding a single, unitless measure of relative inter-muscular mobility. *Adapted from Hojo et al. [15], with modifications.*[3]

[3] ES, erector spinae; L, left; M, multifidus; QL, quadratus lumborum; R, right

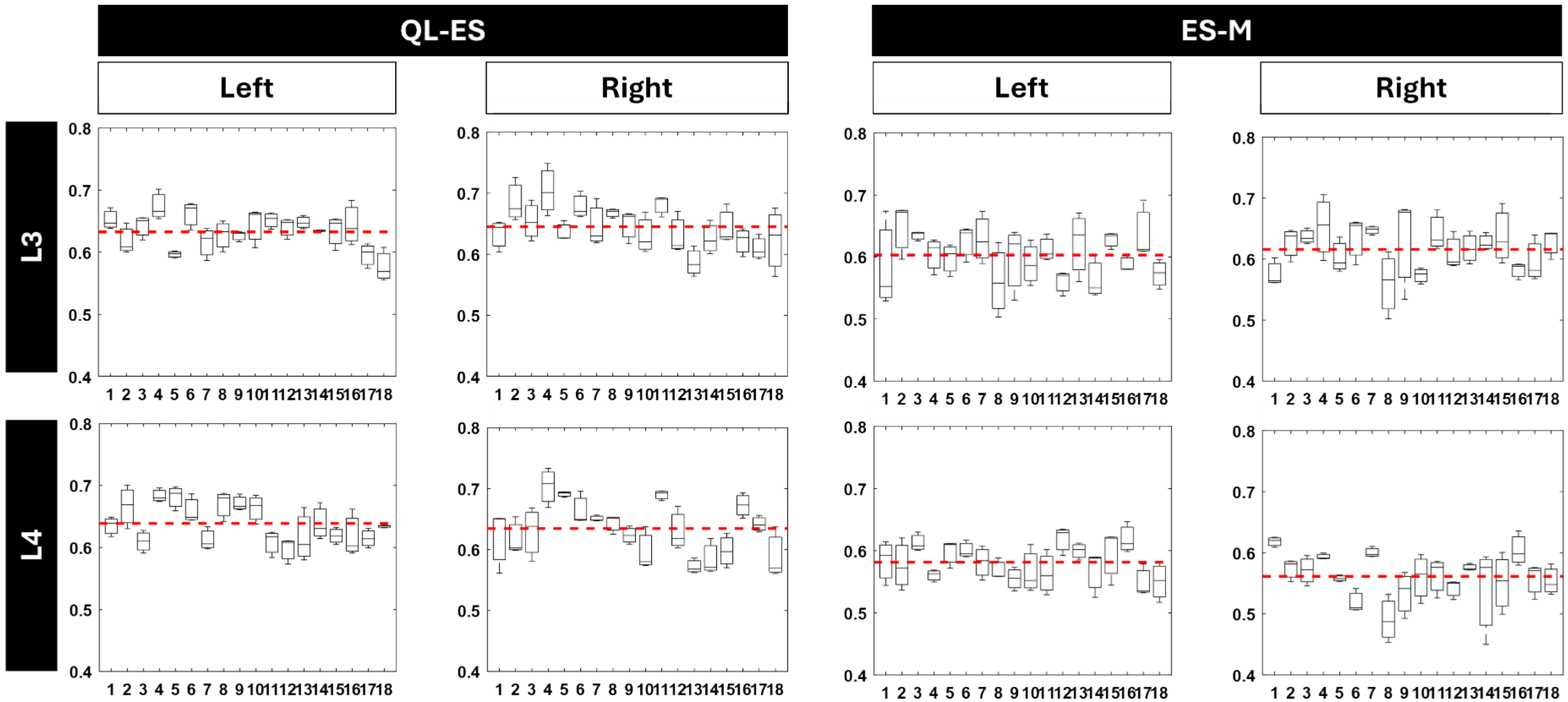

S2: Boxplot of the Dnorm mobility index from three exams for all subjects (n=18) at the quadratus lumborum-erector spinae and erector spinae-multifidus interfaces, at L3 and L4 levels, on the left (the first and third columns) and right (the second and fourth columns) side respectively. The red dotted lines indicate the mean values of the Dnorm mobility index across all subjects. Note: Data from Exam 3 for subject 9 was excluded, so the results reflect only Exams 1 and 2.[4]

[4] ES, erector spinae; M, multifidus; QL, quadratus lumborum

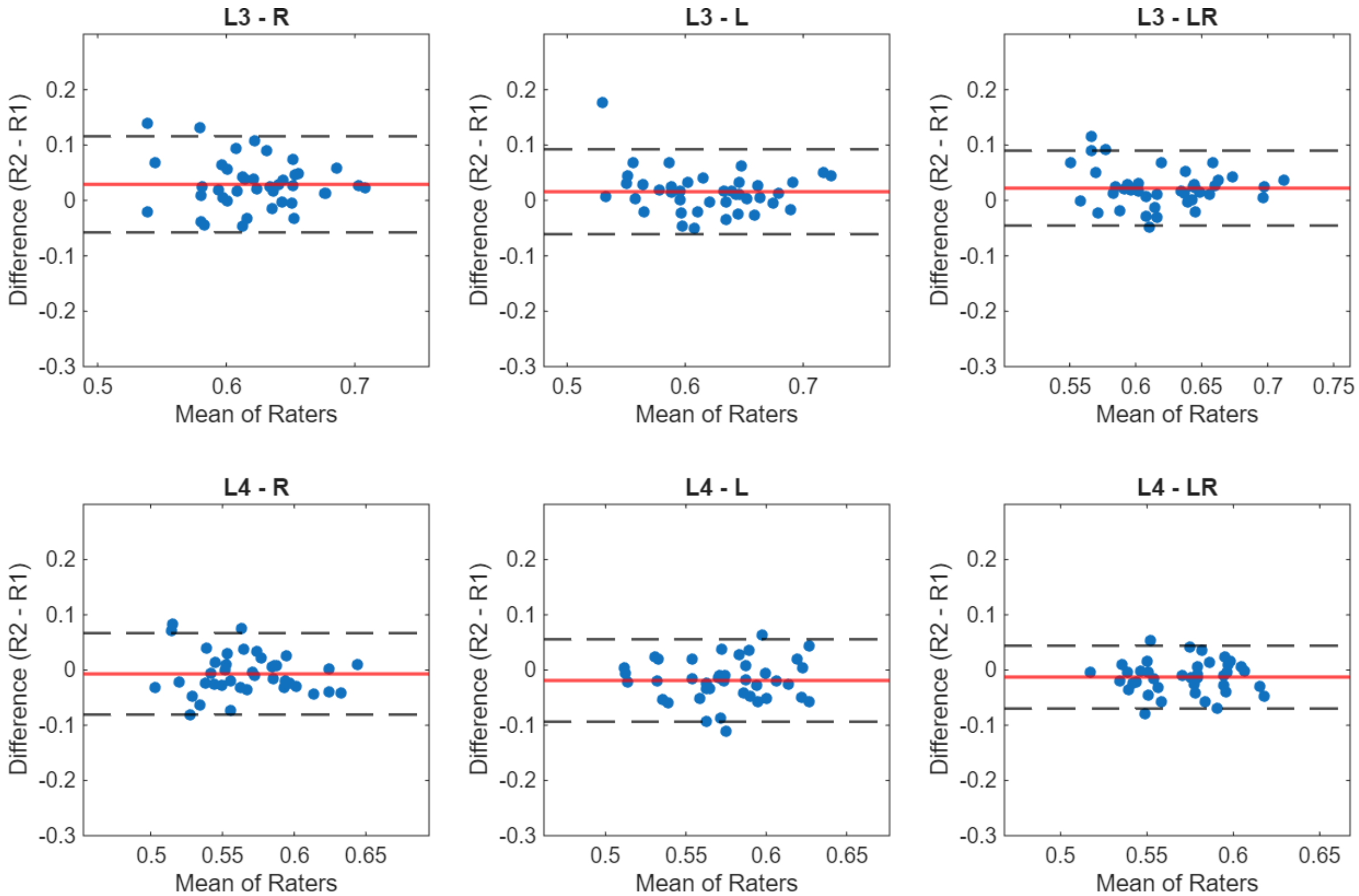

S3: Bland-Altman plots for inter-rater reproducibility of the Dnorm mobility index at the L3 (top row) and L4 (bottom row) erector spinae-multifidus interfaces for the right, left, and bilateral average. Blue dots represent the differences between the two raters (R2 - R1), plotted against the mean of the two raters. The red solid line indicates the mean difference (bias), and the black dashed lines indicate the upper and lower limits of agreement.

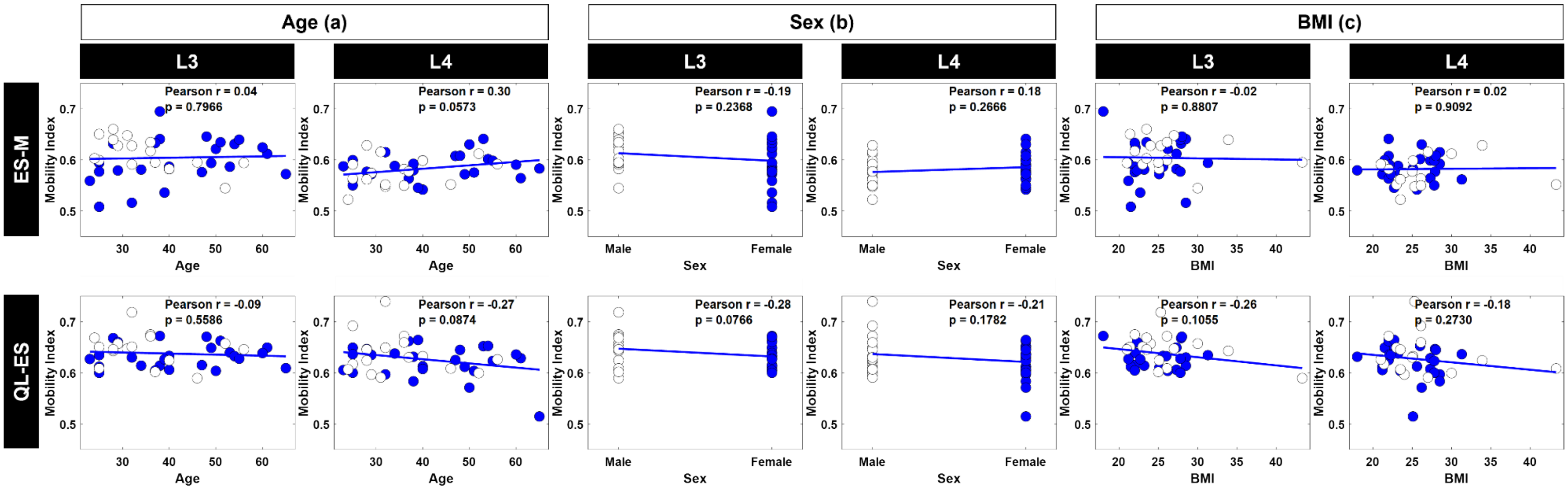

S4: Correlations between mobility index (vertical axis) and Age (**a**), Sex (**b**), and BMI (**c**) (horizontal axes) in HV (n=41) at L3 and L4 for the quadratus lumborum-erector spinae and erector spinae-multifidus. White and blue dots indicate male and female individuals, respectively. Blue lines show linear regression fits. Pearson's *r* and *p*-values are reported in each panel.[5]

[5] BMI, body mass index; ES, erector spinae; M, multifidus; QL, quadratus lumborum

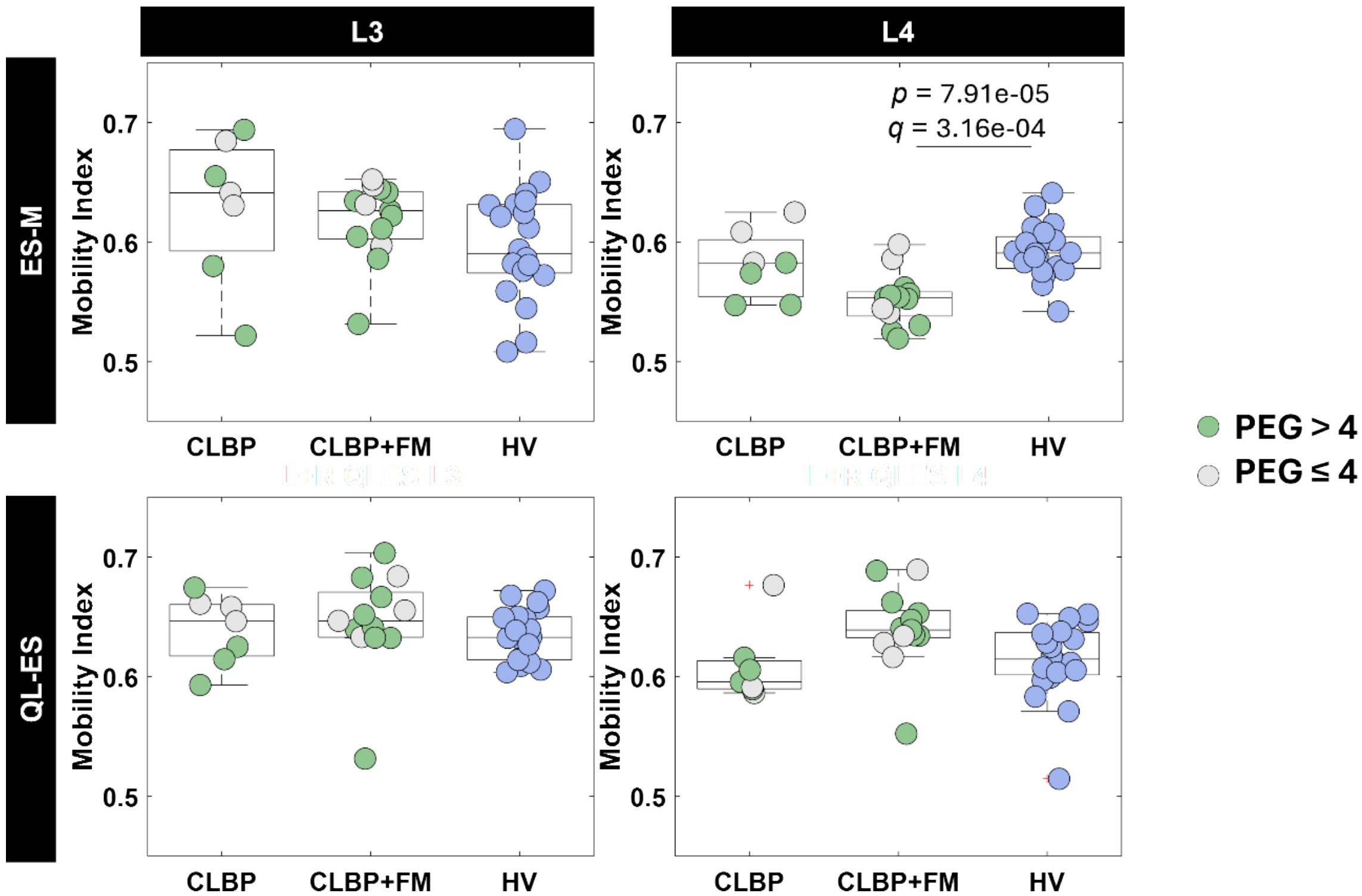

S5: Box plots of mobility indices at the L3 and L4 erector spinae-multifidus and quadratus lumborum-erector spinae interfaces across three groups: chronic low back pain patients with myofascial pain features without or with fibromyalgia and healthy volunteers (blue symbols). Green and grey symbols denote patients with moderate-to-severe pain (PEG > 4) and with mild pain (PEG ≤ 4), respectively. Statistical significance is indicated by corresponding *p* and *q* values.[6]

[6] CLBP-MP, chronic low back pain with myofascial pain features; ES, erector spinae; FM, fibromyalgia; HV, healthy volunteers; M, multifidus; PEG, Pain, Enjoyment, and General Activity; QL, quadratus lumborum

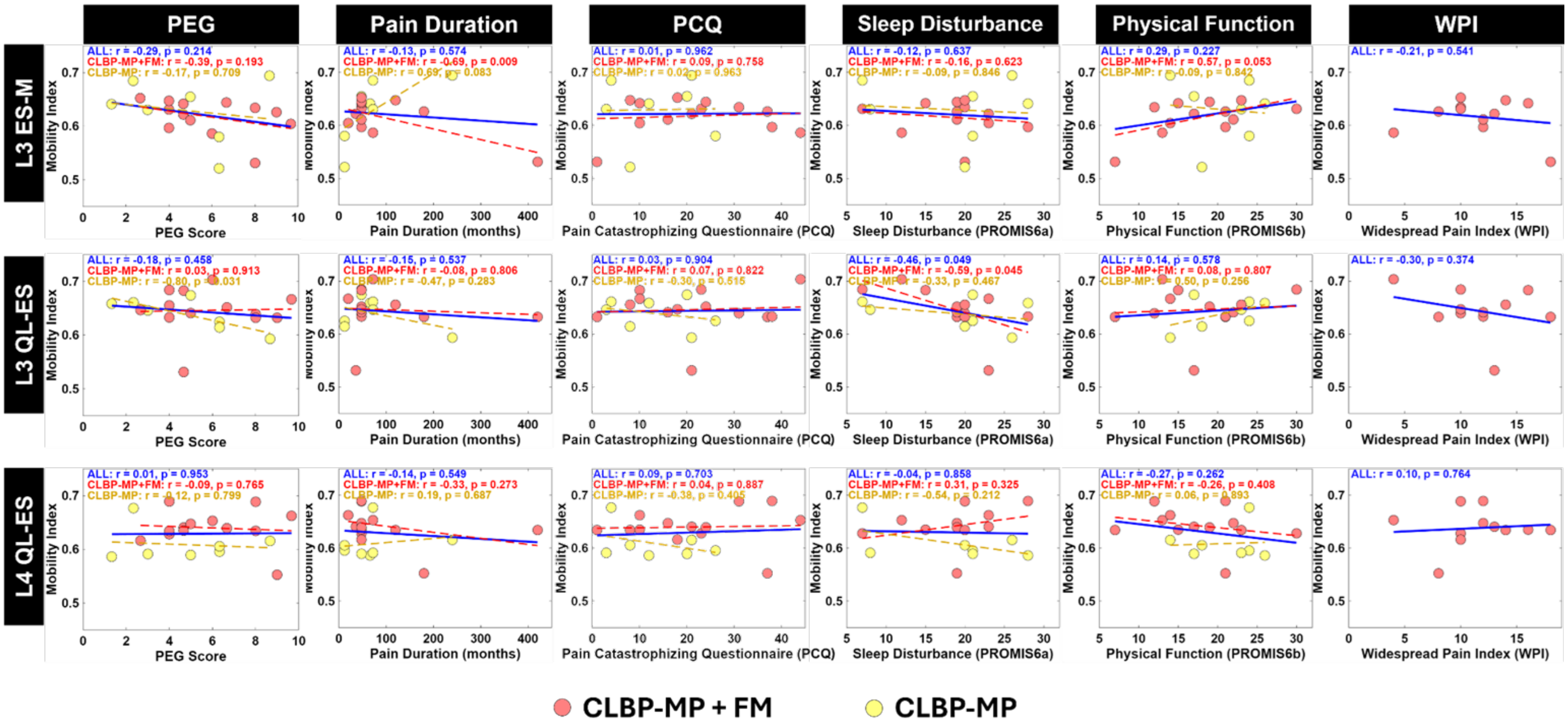

S6: Correlations between mobility index (vertical axis) and six clinical measures (horizontal axis): PEG score, pain duration, Pain Catastrophizing Questionnaire, Sleep Disturbance, Physical Function, and Widespread Pain Index. Columns 1-6 show these measures across patients for L3 erector spinae-multifidus, L3 and L4 quadratus lumborum-erector spinae (rows 1-3). Regression fits are shown for all patients (blue) and for patients with (red) and without (yellow) fibromyalgia. Pearson's *r* and corresponding *p*-values are shown in each panel. Red and yellow plots represent patients with chronic low back pain with myofascial pain features, with and without FM, respectively.[7]

[7] CLBP-MP, chronic low back pain with myofascial pain features; FM, fibromyalgia; PCQ, Pain Catastrophizing Questionnaire; PEG, Pain, Enjoyment, and General Activity; PROMIS-6a, Patient-Reported Outcomes Measurement Information System Sleep Disturbance short form; PROMIS-6b, Patient-Reported Outcomes Measurement Information System Physical Function short form; WPI, Widespread Pain Index.
